# Incidence of Co-Trimoxazole-Induced Hyperkalemia in a Tertiary Care Hospital

**DOI:** 10.2147/RMHP.S283471

**Published:** 2021-02-11

**Authors:** Rana M Al AdAwi, Zainab Albu-Mahmood, Mohamed Abdelgelil, Hani Abdelaziz, Derek Stewart, Ahmed Awaisu

**Affiliations:** 1 Clinical Pharmacist, Hamad General Hospital, Hamad Medical Corporation, Doha, Qatar; 2 Clinical Pharmacist Supervisor, Al Wakra Hospital, Hamad Medical Corporation, Doha, Qatar; 3 Department of Clinical Pharmacy and Practice, College of Pharmacy, QU Health, Doha, Qatar

**Keywords:** co-trimoxazole, co-administration, hyperkalemia, risk factors, adverse drug reaction

## Abstract

**Background:**

Co-trimoxazole is a broad-spectrum antibiotic associated with hyperkalemia.

**Objective:**

To determine the incidence of hyperkalemia and its risk factors in patients receiving co-trimoxazole.

**Materials and Methods:**

A retrospective observational study involving all patients who received co-trimoxazole between 1 January 2012 and 1 January 2013 was conducted. Subjects were identified through a list generated from a computerized pharmacy system. The patients’ demographic and clinical characteristics were retrieved from electronic medical records. Data were analyzed using univariate and multivariate logistic regression.

**Results:**

One hundred sixty-one patients fulfilled the eligibility criteria. Of these, 46 (28.6%) experienced hyperkalemia. Around 35 (76%) of the patients who experienced hyperkalemia received co-administered medications that might induce hyperkalemia. The co-administration of co-trimoxazole with other medications that may induce hyperkalemia was found to be associated with higher incidence of hyperkalemia when compared to co-trimoxazole administration alone [adjusted OR 3.2, 95% CI (1.4–7.3), p=0.005]. Additionally, age > 60 years was associated with an increased risk of hyperkalemia when compared to younger age group 18–39 years [adjusted OR 6.5, 95% CI (2.1–19.7); p=0.001].

**Conclusion:**

Co-trimoxazole use is associated with high incidence of hyperkalemia, especially among older patients and those receiving it in combination with other medications that might contribute to hyperkalemia development such as calcineurin inhibitors and β-blockers.

## Introduction

Co-trimoxazole, a broad-spectrum antibiotic comprising trimethoprim and sulfamethoxazole, is widely used for the treatment and prophylaxis of Gram-positive bacteria, Gram-negative bacteria, and some parasitic infections.^1^ However, co-trimoxazole use has been associated with hyperkalemia, a severe and potentially fatal adverse drug reaction (ADR).^2^,^3^ A study reported that more than half (53%) of the patients with acquired immunodeficiency syndrome (AIDS) who were treated with co-trimoxazole for pneumocystis jiroveci infection developed hyperkalemia.^4^ In addition, emerging evidence suggests that co-trimoxazole can precipitate a life-threatening condition called BRaSh syndrome. It is a combination of bradycardia, renal impairment, drug-induced AV blockage, shock, and hyperkalemia.^5^ The proposed mechanism of co-trimoxazole-induced hyperkalemia is mediated through the blockage of amiloride-sensitive sodium channels in the distal tubule, due to the structural similarity of trimethoprim with potassium-sparing diuretics, leading to the impairment of renal potassium excretion.^2^ Age and co-administered medications have been recognized as risk factors for co-trimoxazole-induced hyperkalemia.^3^ Evidence suggests that concurrent administration of drugs such as angiotensin-converting enzyme inhibitors (ACE-Is), angiotensin-II receptor blockers (ARBs), β-blockers, and spironolactone can increase serum potassium concentration, leading to increased incidence of severe hyperkalemia, hospitalization, and mortality.^3^ ACE-Is, ARBs, and spironolactone have an additional mechanism of inducing hyperkalemia by decreasing the serum concentration of aldosterone, resulting in reabsorbing potassium at the distal renal tubule.^6^ Several studies have also identified age as a factor potentially increasing the risk of hyperkalemia and mortality.^3^ When potassium serum concentration reaches 6 mEq/L or more, treatment interruption and close monitoring are warranted to avoid cardiac toxicity.^2^ Limiting potassium intake and avoiding other medications that might contribute to hyperkalemia are approaches to consider when the potassium level is controlled below 6 mEq/L.^2^ Despite the clinical significance of co-trimoxazole-induced hyperkalemia, this ADR has been neglected by many clinicians, and no clear guidelines are available to monitor patients.

## Aim of the Study

This study aimed to investigate the incidence of hyperkalemia among patients who received co-trimoxazole and had normal serum potassium concentrations at baseline and to identify potential risk factors for the hyperkalemia.

### Materials and Methods Study Design

This was a retrospective observational study of patients who received co-trimoxazole for therapeutic or prophylactic indications at Hamad General Hospital (HGH) in Qatar. The therapeutic indications included treatment of acute spontaneous bacterial peritonitis (SBP), mild diabetic foot infection, toxoplasmosis, urinary tract infection, and Pneumocystis pneumonia. The prophylactic indications included bite wound infection prophylaxis, SBP long-term secondary prevention, and Pneumocystis pneumonia prevention in immunocompromised patients. Patients were identified through a computerized pharmacy system, via an automated report generated between 1 January 2012 and 1 January 2013. Baseline potassium serum level was considered as any level within one week prior to initiation of co-trimoxazole; all subsequent levels were follow-up. Hyperkalemia was defined as serum potassium level above 5.5 mEq/L.^2^ Patients’ electronic medical records were reviewed to obtain the laboratory parameters (serum creatinine and serum potassium levels at baseline and follow-up). Patients’ medication histories were reviewed to identify any co-administered medications that has the potential to induce hyperkalemia.

### Ethics Approval

This study was reviewed and approved by the Institutional Review Board (IRB) of the Medical Research Center at Hamad Medical Corporation (HMC's MRC) in Qatar (approval number: #14425/14). This is a retrospective observational chart review and obtaining informed consent to participate in the study from the research subjects was not applicable. In such cases, the IRB typically grants a waiver of consent. Further, all patient data accessed complied with relevant data protection and privacy regulations in Qatar.

### Eligibility Criteria

Adult patients (≥18 years) with normal renal function (GFR ≥60 mL/min/1.73m^2^) who received co-trimoxazole during the study period and had documented potassium serum levels at baseline and during follow-up were included. Patients who received less than two doses of co-trimoxazole and/or had no potassium follow-up were excluded from the study.

### Data Collection

Electronic medical records were reviewed to obtain relevant data at baseline and follow-up: demographics (age and sex); dose, frequency, and duration of co-trimoxazole; comorbid conditions, and concurrent medications potentially affecting potassium serum levels including β-blockers (selective and non-selective), calcineurin inhibitors, heparin, ACE-I, ARBs, potassium-sparing diuretics (spironolactone, eplerenone), nonsteroidal anti-inflammatory drugs (NSAIDs), and antifungals (fluconazole). In addition, relevant laboratory data like serum potassium and serum creatinine concentrations were collected. Baseline potassium and creatinine levels were considered as any level within one week prior to initiation of co-trimoxazole. Any potassium or creatinine level within the first week of the short-treatment course or within one month of the long-term prophylactic indications were considered as follow-up readings.

### Data Analysis

Data were analyzed using SPSS version 18 (IBM SPSS^®^ Statistics for Windows; IBM Corp, Armonk, New York, USA). Both descriptive and inferential statistics were applied for the data analysis. The 95% confidence intervals (CIs) for each mean were also calculated. Chi-square was used to compare the incidence of hyperkalemia between different factors. Univariate and multivariate logistic regression analyses were conducted to determine the association between risk factors and development of co-trimoxazole-induced hyperkalemia and compute adjusted and unadjusted odds ratios (ORs). The factors included in the multivariate logistic regression analysis were age, gender, total daily dose [the once and twice daily doses of the double strength (DS) tablet 800/160 mg], and the co-administered medications. The level of statistical significance was defined as p≤ 0.05.

## Results

**Figure 1:**
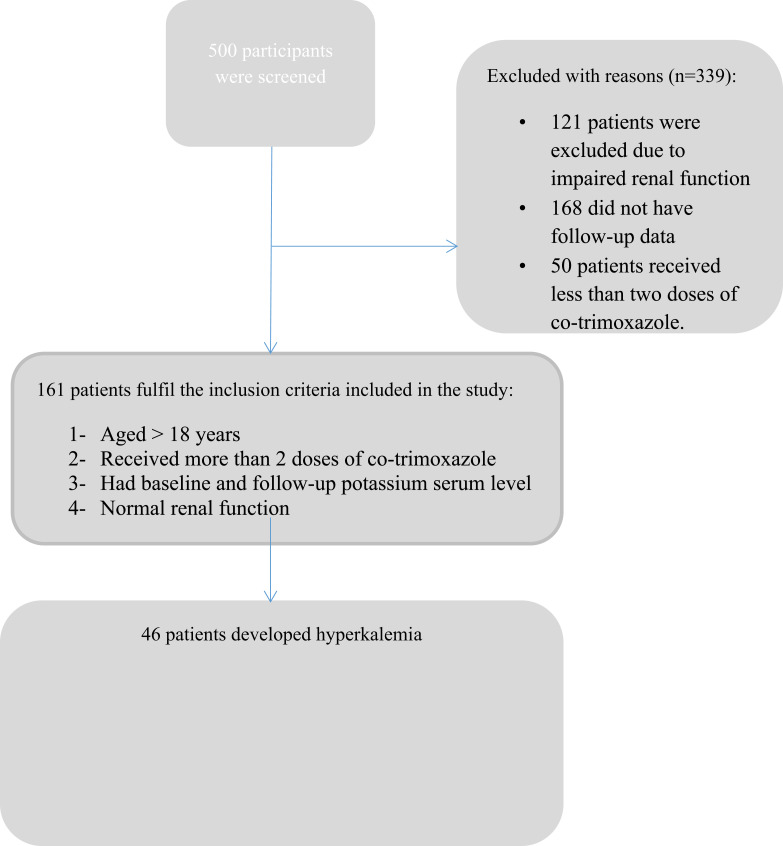
A flow chart of the screening process.

Of the 500 patients reviewed, 121 patients were excluded due to impaired renal function (GFR <60 mL/min/1.73 m^2^), 168 did not have follow-up data, and 50 patients received less than two doses of co-trimoxazole. One hundred sixty-one patients who fulfilled the inclusion criteria were included (Figure 1). Of these, 57.1% (n=92) were male and 57.1% (n=92) were prescribed other medications known to contribute to hyperkalemia. Around 24% of the patients had hypertension, 56% had diabetes, and 46% were status-post organ transplant. The patients were divided into two groups based on the co-trimoxazole (DS tablet 800/160 mg) dosing frequency; once daily vs twice daily dosing regimen. The number of patients in each group was comparable (Table 1).

**Table 1:** Incidence of Hyperkalemia in Patients Receiving Co-Trimoxazole (n = 161)

	Total (n=161)	Once Daily Dosing (n=75)	Twice Daily Dosing (n=86)	
Hyperkalemia in the entire population regardless of the interacting drugs	46 (28.6%)	21 (28%)	25 (29%)	
Hyperkalemia in patients receiving co-trimoxazole without interacting drugs	11 (24%)	7 (33%)	4 (16%)	
Hyperkalemia in patients receiving co-trimoxazole with other medications that are known to influence potassium level	35 (76%)	14 (67%)	21 (84%)	
Incidence of hyperkalemia among different co-administered medication groups				
	**Total (n=35)**	**Once Daily (n=14)**		**Twice Daily (n=21)**
*β-blocker	8 (22.9%)	2 (14.3%)		6 (28.6%)
**Immunosuppressant agent + β-blocker	9 (25.7%)	3(21.4%)		6 (28.6%)
Heparin	3 (8.6%)	3 (21.4%)		0 (0%)
***ACEIs/ARBs	3 (8.6%)	2 (14.3%)		1 (4.8%)
** Calcineurin inhibitors	12 (34.3%)	4 (28.6%)		8 (38.1%)

**Notes:** *β-blocker: bisoprolol, metoprolol, carvedilol, atenolol, propranolol. ** Calcineurin inhibitors: cyclosporine, tacrolimus. ***ACEIs/ARBs: Lisinopril, enalapril, irbesartan, valsartan, losartan.

Hyperkalemia (potassium serum concentration >5.5 mEq/L) was observed in 46 (28.6%) patients, of whom, 25/46 (54.3%) were in the twice-daily dosing group. Thirty-five (76%) of the patients who developed hyperkalemia were receiving concurrent medications known to increase potassium serum levels (Table 1). Receiving co-trimoxazole in combination with β-blockers, calcineurin inhibitors alone or in combination with β-blockers was significantly associated with higher incidence of hyperkalemia when compared to receiving co-trimoxazole alone [OR 3.2, 95% CI (1.1–9.7), p=0.035, OR 3.2, 95% CI (1.2–8.3), p=0.019, OR 3.4, 95% CI (1.2–9.8), p=0.024] (Table 2). Both selective and non-selective β-blockers were considered in the analysis [40 patients received selective β -blockers (atenolol, metoprolol, or bisoprolol) and 13 received the non-selective ones (propranolol or carvedilol)]. Of note, during the study period, no patients were identified to co-administer potassium-sparing diuretics, NSAIDs, or potassium supplements with the co-trimoxazole. Moreover, patients with age >60 years were more prone to develop hyperkalemia when compared to younger age groups 18–39 years [OR 6.37, 95% CI (2.6–18.8), p=0.001]

**Table 2:** Univariate Logistic Regression Analysis for Factors Associated with Hyperkalemia in Patients Receiving Co-Trimoxazole

Variables	Percentage of Hyperkalemia (n)	Unadjusted OR	95% CI	p-value
Age				
18−39	10.2% (5)	1		
40–60	32.3% (20)	4.19	(1.4–12.2)	0.009
>60	42% (21)	6.37	(2.6–18.8)	0.001
Sex				
Female	26.1% (18)	1	(0.6–2.5)	0.5
Male	30.4% (28)	1.24		
Dosing frequency				
Once daily (n=75)	28% (21)	0.95	(0.5–1.9)	0.9
Twice daily (n=86)	29% (25)	1		
Taking other medications listed below				
Yes (n=92)	38% (35)	3.2	(1.5–7)	0.003
No (n=69)	15.9% (11)	1		
β-blocker alone				
Yes (n=21)	38% (8)	3.2	(1.1–9.7)	0.035
No (n=69)	15.9% (11)	1		
ACEIs & ARBs				
Yes (n=10)	30% (3)	2.3	(0.5–10.1)	0.3
No (n=69)	15.9% (11)	1		
Heparin				
Yes (n=6)	50% (3)	5.3	(0.9–29.6)	0.059
No (n=69)	15.9% (11)	1		
Immunosuppressant agent alone				
Yes (n=32)	37.5% (12)	3.2	(1.2–8.3)	0.019
No (n=69)	15.9% (11)	1		
Immunosuppressant agent + β-blocker				
Yes (n=23)	39.1% (9)	3.4	(1.2–9.8)	0.024
No (n=69)	15.9% (11)	1		

After adjusting the confounders with the multivariate logistic regression analysis, only age and co-administered medications were found to be significant. Patients with age >60 years were more prone to develop hyperkalemia when compared to the younger age group 18–39 years [adjusted OR 6.5, 95% CI (2.1–19.7), p=0.001]. Moreover, the middle age group (40–60 years) was also found to have higher incidence of hyperkalemia than the younger age group 18–39 years [adjusted OR 3.5, 95% CI (1.2–10.5), p=0.023]. In addition, the co-administration of other medications that may induce hyperkalemia was found to be associated with higher incidence of hyperkalemia when compared to those receiving co-trimoxazole alone [adjusted OR 3.2, 95% CI (1.4–7.3), p=0.005].

## Discussion

Almost one-third of the patients receiving co-trimaxazole developed hyperkalemia, with age >60 years identified as high-risk. In addition, co-administration of medications that are known to raise potassium serum level increased the risk of hyperkalemia by three folds [adjusted OR 3.2, 95% CI (1.4–7.3), p=0.005]. However, dosing frequency did not significantly influence the hyperkalemia incidence.

A previous study by Fralick et al affirmed that co-trimoxazole use with an ACE-I or ARB was associated with increased risk of sudden death due to severe hyperkalemia [OR 1.38: 95% CI (1.09 to 1.76)] when compared with amoxicillin.^3^ In our study, the incidence of hyperkalemia was 3-fold higher amongst patients who received medications that increase serum potassium level. However, the incidence of hyperkalemia amongst patients who received ACE-Is or ARBs vs those who received co-trimoxazole alone was 8.6% (n=3) vs 15.9% (n=11) (Tables 1 and 2). The small group of patients who received ACE-Is/ARBs (n=16 out 161) could be the reason for the deviation from published literature. Furthermore, among the β-blocker users, Weir et al found that the rate of hospitalization among co-trimoxazole users was considerably higher when compared to amoxicillin (OR 5.1; 95% CI 2.8 to 9.4).^8^ Evidence indicates that non-selective β-blockers are more commonly associated with hyperkalemia than the cardioselective ones.^9^ However, there are some evidence that suggested the potential of selective β-blockers like metoprolol,^10^,^11^ bisoprolol,^12^ and atenolol^13^ to induce hyperkalemia.^11^,^12^ In the current study, both selective and non-selective β-blockers were included, where 38% (8) of β-blocker users developed hyperkalemia.

A recent cohort study showed a 0.28 mmol/l (95% CI 0.03–0.53, p=0.031) increase in serum potassium level was associated with co-trimoxazole use compared to ceftriaxone use. It is noteworthy that the population of the study was older adults [mean age 73.8 (12.5) years]; hence, the incidence of hyperkalemia was more pronounced.^14^ A post-hoc analysis of a randomized controlled trial showed that amongst older patients [mean age 72 (8.5) years old], co-trimoxazole raised the mean potassium level by 0.21 mmol/l at 6 weeks treatment time when compared to placebo.^15^ Additionally, a nested case–control study of a cohort of older patients (>66 years) concluded that the potential for hyperkalemia-associated hospitalization was increased seven-fold among older patients receiving ACE-Is or ARBs (adjusted OR, 6.7; 95% CI, 4.5–10.0).^8^ Studies have also demonstrated that older people receiving co-trimoxazole in combination with other medications known to contribute to hyperkalemia are at higher risk of hyperkalemia,^3^,^7^ which is consistent with our findings that advanced age was associated with higher risk of hyperkalemia.

In this study, the co-administration of calcineurin inhibitors, either alone or in combination with β-blockers, was significantly associated with increased incidence of hyperkalemia. Several studies and case reports found that tacrolimus and cyclosporine cause elevation in plasma potassium concentration, which can be further augmented by co-administration of an ACE-I or an ARB.^5^,^16^

A review by Perazella et al reported that co-trimoxazole can cause hyperkalemia regardless of dose.^2^ However, a retrospective study showed that hyperkalemia incidence was more frequent with the higher doses of co-trimoxazole.^17^ In this study, the results failed to confirm a correlation between hyperkalemia incidence and the total daily doses of co-trimoxazole (once daily or twice daily doses of the DS 800/160 mg).

There is some evidence that pharmacists have a crucial role in monitoring drug therapy to detect and prevent ADRs.^18^ Hence, pharmacists can contribute to active monitoring of potassium levels and additionally educating patients on the potential for interactions with other medication, conditions, and food.

This study has the inherent limitations of a retrospective study design in that only the specific data collected and recorded during routine clinical practice could be captured. While we excluded those with renal impairment, hyperkalemia could be a result of other factors such as excess dietary potassium intake, renal and adrenal insufficiencies, and other factors. The limited sample size may have meant that the study was underpowered for the comparison made; hence, the results should be interpreted with caution. There may also be issues with generalizability to other populations and settings. Furthermore, the study was completed several years earlier, although clinical practice is relatively unchanged hence the data and findings remain valid. There is a need for prospective studies with adequate sample sizes and more frequent monitoring of potassium and serum creatinine levels.

## Conclusion

Co-trimoxazole administration was associated with an increased risk of hyperkalemia especially among older patients and those receiving other co-administered medications that may induce hyperkalemia. Caution should be exercised when co-trimoxazole is used in older people and patients receiving other medications affecting potassium levels such as calcineurin inhibitors and the combination of calcineurin inhibitors and β-blockers.
